# Functional genomic profiling of O-GlcNAc reveals its context-specific interplay with RNA polymerase II

**DOI:** 10.1186/s13059-025-03537-2

**Published:** 2025-03-24

**Authors:** Sofia Rucli, Nicolas Descostes, Yulia Ermakova, Urvashi Chitnavis, Jeanne Couturier, Ana Boskovic, Matthieu Boulard

**Affiliations:** 1 Epigenetics & Neurobiology Unit, European Molecular Biology Laboratory, EMBL Rome, Rome, Italy; 2 Collaboration for a joint PhD degree between EMBL and Heidelberg University, Heidelberg, Germany

**Keywords:** O-linked β-N-acetylglucosamine, O-GlcNAc, OGT, Glycosylation, Transcription, Promoter, RNA polymerase II, Degron, CUT&RUN, ChIP-seq, RNA-seq, ChIP‐Atlas

## Abstract

**Background:**

How reversible glycosylation of DNA-bound proteins acts on transcription remains scarcely understood. O-linked β-N-acetylglucosamine (O-GlcNAc) is the only known form of glycosylation modifying nuclear proteins, including RNA polymerase II (RNA Pol II) and many transcription factors. Yet, the regulatory function of the O-GlcNAc modification in mammalian chromatin remains unclear.

**Results:**

Here, we combine genome-wide profiling of O-GlcNAc-modified proteins with perturbations of intracellular glycosylation, RNA Pol II-degron, and super-resolution microscopy. Genomic profiling of O-GlcNAc-modified proteins shows a non-random distribution across the genome, with high densities in heterochromatin regions as well as on actively transcribed gene promoters. Large-scale intersection of the O-GlcNAc signal at promoters with public ChIP-seq datasets identifies a high overlap with RNA Pol II and specific cofactors. Knockdown of *O-GlcNAc Transferase* (*Ogt*) shows that most direct target genes are downregulated, supporting a global positive role of O-GlcNAc on the transcription of cellular genes. Rapid degradation of RNA Pol II results in the decrease of the O-GlcNAc levels at promoters encoding transcription factors and DNA modifying enzymes. RNA Pol II depletion also unexpectedly causes an increase of O-GlcNAc levels at a set of promoters encoding for the transcription machinery.

**Conclusions:**

This study provides a deconvoluted genomic profiling of O-GlcNAc-modified proteins in murine and human cells. Perturbations of O-GlcNAc or RNA Pol II uncover a context-specific reciprocal functional interplay between the transcription machinery and the O-GlcNAc modification.

**Supplementary Information:**

The online version contains supplementary material available at 10.1186/s13059-025-03537-2.

## Background

The chromatin of virtually all mammalian cells is bound by a substantial number of glycosylated proteins. In the nucleus, the only known form of glycosylation is the linkage of the monosaccharide O-linked β-N-acetylglucosamine (O-GlcNAc) to specific serine (Ser) and threonine (Thr) hydroxyls [1]. Contrary to the glycosylation of membrane and secreted proteins, intracellular O-GlcNAc glycosylation is reversible. O-GlcNAc’s homeostasis is controlled by two highly conserved enzymes: the O-GlcNAc transferase (OGT) that adds the O-GlcNAc post-translational modification (PTM), and the O-GlcNAcase (OGA) that removes it [2]. The donor substrate UDP-GlcNAc is the end product of the hexosamine biosynthetic pathway which integrates inputs from glucose, amino acids (glutamine), acetyl-CoA, and nucleotide metabolism [3]. Hence, O-GlcNAc is widely regarded as a metabolic-sensitive PTM involved in the regulation of protein stability [4], degradation [5], folding [2], and localization [3].

To date, over 5000 intracellular proteins were found to be modified by O-GlcNAc [1]. This number is almost certainly underestimated due to the technical challenge of detecting O-GlcNAc in biological samples [6]. The lability of the glycosidic bond required the usage of electron-transfer dissociation (ETD) as a mass spectrometry method for direct detection of O-GlcNAc [7]. Applying this method to murine embryonic stem cell (ESC) nuclei, O-GlcNAc was mapped on histone modifying enzymes (EP400, KDM3B, JMJD1C), the ten-eleven translocation methylcytosine dioxygenases (TET1,2,3), DNA methyltransferase I (DNMT1), some transcriptional repressors (SPEN and SIN3A) and a plethora of transcription factors (TFs), including the pluripotency master regulators SOX2, OCT4 and KLF4 [8–11]. The list of O-GlcNAc sites on nuclear and cytosolic proteins is expanding thanks to recent progress in enrichment methods and mass spectrometry [1, 7, 12]. Based on indirect evidence, histones were proposed to be O-GlcNAc modified [13, 14], but skepticism has grown due to the lack of reproducibility of this finding [6, 15].

The biological role of the O-GlcNAc modification in mammals remains scarcely understood. This gap of knowledge stems from the pleiotropy of this modification, its essential nature, and the lack of molecular probes, such as antibodies, to detect the O-GlcNAcylated form of specific proteins. Genetic studies in Drosophila uncovered an essential function for *Ogt* (also known as *super sex comb*) in Polycomb-mediated transcriptional silencing of homeotic genes and other developmental genes [16]. Mechanistically, the O-GlcNAc modification of the repressor Polyhomeotic is crucial to prevent its non-productive aggregation [17]. It has been difficult to assess whether this function is conserved in mammals because *Ogt* is an essential (X-linked) gene for the cellular viability of all examined cell types [18, 19]. Using different genetic strategies in the mouse to perturb O-GlcNAc, we and others previously reported that the O-GlcNAc modification is required for the stable silencing of retrotransposons [19–22].

RNA polymerase II (RNA Pol II) was among the first proteins to be found O-GlcNAc modified [23]. The carboxyl-terminal domain (CTD) of human and murine RNA Pol II is composed of 52 repeats of the heptapeptide motif Y_1_S_2_P_3_T_4_S_5_P_6_S_7_. O-GlcNAc was mapped by mass spectrometry at Ser2, Thr4 and Ser5 [23, 24]. Two main different RNA Pol II states were identified, the phosphorylated and the O-GlcNAcylated forms, and these two forms were shown to be mutually exclusive [23]. The RNA Pol II CTD sequence undergoes a dynamic cycle of several PTMs that precisely coordinate RNA Pol II activity and transcriptional progression [25]. Chemical inhibition of OGT’s activity was shown to prevent RNA Pol II recruitment to the promoter region [24]; moreover, inhibition of either OGT or OGA interferes with the assembly of the pre-initiation complex (PIC), an indispensable step for transcription initiation [26].

Thus far, mechanistic studies interrogating the role of O-GlcNAc on RNA Pol II have been performed in vitro on reconstituted biochemical systems [24, 27]. Hence, the influence of genomic contexts on the local chromatin glycosylation, and its transcriptional consequences remain unexplored.

Here, we investigated the regulatory role of O-GlcNAc genome-wide through a combination of functional assays in murine embryonic stem cells (ESCs) and in human colorectal adenocarcinoma cells (DLD-1). We detected a high occupancy of O-GlcNAc-modified proteins at some actively transcribed gene promoters in addition to repressed retrotransposons. Large-scale integrated ChIP-seq analysis revealed that RNA Pol II and some specific associated factors are the most likely candidates to be modified by O-GlcNAc at active gene promoters. Functionally, O-GlcNAc depletion caused significant changes in the expression of hundreds of genes and resulted in the downregulation of O-GlcNAc’s direct targets, supporting a role in enhancing transcription. Rapid depletion of RNA Pol II resulted in higher O-GlcNAc levels at a set of promoters encoding for the transcriptional machinery and lower O-GlcNAc levels at another set of promoters encoding DNA repair enzymes, thus uncovering a context-specific RNA Pol II dependency of O-GlcNAc modification.

## Results

### O-GlcNAc-modified proteins occupy heterochromatin regions and transcribed promoters

**Fig. 1:**
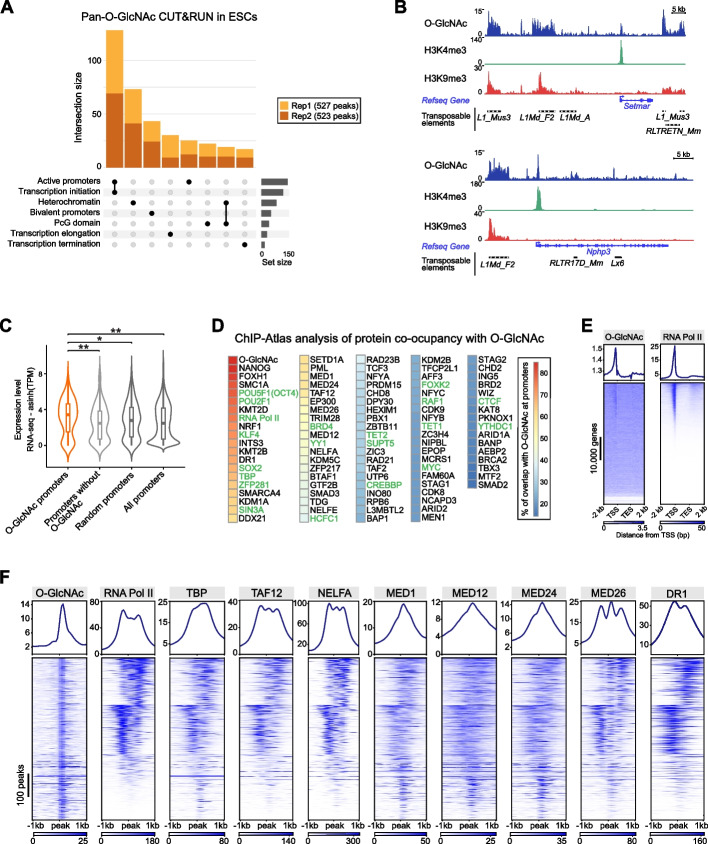
O-GlcNAc-modified proteins occupy heterochromatin regions and gene promoters. **A** Upset plot showing the distribution of O-GlcNAc peaks (527 and 523 peaks for replicates 1 and 2) profiled by CUT&RUN in murine ESCs across the following functional genomic regions: Active promoters (79 and 84 peaks), transcription initiation (64 and 71 peaks), heterochromatin (42 and 52 peaks), bivalent promoters (29 and 30 peaks), Polycomb (PcG) domains (24 and 23 peaks), transcription elongation (23 and 12 peaks), and transcription termination (9 and 11 peaks). Two technical replicates are shown. **B** Representative genomic tracks of O-GlcNAc occupancy patterns in correlation with H3K4me3 (Sequence Read Archive (SRA): SRX5382140 [80]) as a marker of gene promoter and H3K9me3 (SRA: SRR925652 [81]) as a marker of heterochromatin regions. At the bottom, Refseq genes, LINE1 (L1), and LTR retrotransposons are shown. **C** Violin plot showing the expression levels of genes using a publicly available RNA-seq (SRA: SRR11294181 [31]) with promoters highly occupied by O-GlcNAc (CUT&RUN) modified proteins (*n* = 236), promoters without detectable O-GlcNAc signal (*n* = 236), randomly selected promoters (*n* = 236), and all promoters (*n* = 21,085). The boundaries of the overlaid box plot show the data above the 1st and within the 3.^rd^ quartiles, whiskers indicate minimum and maximum values, and the horizontal bar in the box plot shows the median. Differences in median expression levels were assessed with a Mann–Whitney-Wilcoxon two-sided test. *: *p* < 0.05; **: *p* < 0.01. **D** Percentages of signal overlap at promoters between O-GlcNAc peaks and 21,205 ChIP-Atlas datasets in mouse ESCs. Proteins previously described to be O-GlcNAcylated are indicated in green. The highest overlap is found with the pan-O-GlcNAc ChIP-seq GSE93539. **E** Genes-stack plots showing the O-GlcNAc CUT&RUN signal (left) and RNA Pol II (right) at all Ensembl genes (*n* = 55,634). **F** Heatmap showing O-GlcNAc CUT&RUN signal along with ChIP-seq signal of proteins involved in transcription with high genomic overlap with O-GlcNAc + / − 1 kb around O-GlcNAc peak centers, ordered by k-mean clustering on RNA Pol II signal on the union of the replicate peaks (702 peaks). All rows are centered on O-GlcNAc peaks based on the ranking of signals. The percentages of overlap with each O-GlcNAc replicate are RNA Pol II (72/76%), TBP (68/67%), TAF12 (56/57%), NELFA (45/46%), MED1 (58%), MED12 (49/48%), MED24 (57/58%), MED26 (47/49%), and DR1 (75/69%)

To gain insight into the genomic contexts of glycosylated proteins at the chromatin level, we sought to determine their genome-wide occupancy in ESCs. The genomic profiling of O-GlcNAc-modified proteins, in situ and in native conditions, is required to circumvent the problem of sequestration of anti-O-GlcNAc antibodies by the densely O-GlcNAcylated nuclear pore proteins and complex glycans of the cell membrane. To this end, we modified the CUT&RUN protocol [28] on fractionated nuclei to increase permeabilization. We used the pan-O-GlcNAc monoclonal antibody HGAC85, whose specificity had been previously validated using *Ogt*-null fly larvae [16, 29]. This antibody targets the O-GlcNAc modification itself independently of its protein substrate. Hence, the resulting CUT&RUN signal represents the genomic density of glycosylated proteins, regardless of their identity. Our CUT&RUN analysis revealed that O-GlcNAc-modified proteins are predominantly localized at active promoters and heterochromatin regions (Fig. 1A and Additional file 1: Fig. S1A). The enrichment of O-GlcNAcylated proteins at promoter regions was also confirmed by chromatin immunoprecipitation sequencing (ChIP-seq; Additional file 1: Fig. S1B, C).

We and others previously reported the binding of O-GlcNAc-modified proteins at retrotransposons that are occupied by nucleosomes trimethylated at lysine 9 of histone H3 (H3K9me3) forming heterochromatin domains [20, 21]. In the present study, we focus on the role of O-GlcNAc on proteins associated with cellular genes’ promoters (non-transposable elements).

The enrichment of O-GlcNAc at transcription start sites (TSSs) alongside trimethylation at lysine 4 of histone H3 (H3K4me3) (Fig. 1B) is consistent with previous reports [27, 30]. We next assessed whether there could be a correlation between O-GlcNAc signal at promoters and their expression level using RNA-seq data of wild-type (WT) ESCs grown in the same condition [31]. The median expression of genes occupied by O-GlcNAcylated proteins was higher than those lacking O-GlcNAcylation (*p* = 0.000692, Mann–Whitney-Wilcoxon two-sided test); hence, promoters occupied by O-GlcNAc modified proteins are on average more expressed than those lacking it (Fig. 1C and Additional file 1: Fig. S1D).

The identity of the O-GlcNAc-modified proteins that contribute to the O-GlcNAc signal is unknown. To identify candidates and characterize the local protein context underlying O-GlcNAc peaks, we computationally screened for peaks colocalization at promoters between the merged O-GlcNAc replicates and 21,205 ChIP-seq peak datasets from the ChIP-Atlas database [32]. Among the several chromatin-binding proteins overlapping with O-GlcNAc peaks, many were previously reported to be glycosylated (Fig. 1D and Additional file 2: Table S1) [8–10, 23, 33–42]. These include TET1 and TET2 which were previously shown to share a similar genomic distribution as pan-O-GlcNAc, occupying gene promoters and retrotransposons [22]. The TET enzymes catalyze the oxidation of 5-methylcytosine (5mC) to 5-hydroxymethylcytosine (5hmC). Recent studies found that their O-GlcNAc modification regulates their activity and, in turn, DNA methylation patterns globally [20, 38]. Of note, factors whose ChIP-seq signal greatly overlap with O-GlcNAc can either be O-GlcNAc-modified themselves or alternatively co-occupy loci bound by the former. For example, NANOG, which displays a high overlap with O-GlcNAc (85.5% overlap, Additional file 2: Table S1), was shown to lack O-GlcNAc but co-binds with the O-GlcNAc-modified proteins OCT4, SOX2, and KLF4 (Additional file 1: Fig. S1E) [8, 9].

Over 70% of the O-GlcNAc signal overlapped with RNA Pol II binding sites, as previously reported in mouse bone marrow [30] (Fig. 1D and Additional file 2: Table S1). This result and the previously reported glycosylation of the CTD of RNA Pol II [23, 24] prompted us to examine the density of O-GlcNAc-modified proteins and RNA Pol II occupancy at all gene promoters. Figure 1E shows a strong correlation between RNA Pol II and O-GlcNAc -occupied loci, with a narrower O-GlcNAc signal at almost each locus. This genomic O-GlcNAc pattern could be contributed by RNA Pol II glycosylation as well as by other O-GlcNAc-modified components of the transcription machinery, such as TATA-box binding protein (TBP), which was also found O-GlcNAc modified [23, 35]. Accordingly, several proteins with high co-occupancy with O-GlcNAc are functionally related to RNA Pol II and participate in transcription initiation (mediator subunits [43, 44], TBPs [45, 46]) and negative elongation factor (NELF) complexes [47, 48]; Fig. 1F).

We next investigated whether genomic O-GlcNAc patterns could be responsive to a metabolic stimulus. Because global levels of O-GlcNAcylation were shown to increase upon elevated levels of extracellular glucose [9, 11], we asked whether genomic occupancy of O-GlcNAc-modified proteins could be sensitive to changes in extracellular glucose. We compared O-GlcNAc genomic profiles under high glucose conditions (25 mM, standard ESC culture condition) with cultures in low-glucose (5.5 mM) media for 6 h, 48 h, and 7 days. While modest changes were observed at 48 h (Additional file 1: Fig. S1F), no statistically significant differential occupancy of glycosylated proteins was detected. We cannot exclude, however, that the glucose concentration in ESC culture media is above the threshold of nutrient deprivation at low dose to trigger a change in chromatin O-GlcNAcylation.

Overall, our genomic analysis indicates that in addition to heterochromatin, high density of O-GlcNAc-modified proteins is found at a set of actively transcribed gene promoters and co-localize with RNA Pol II and some specific associated factors.

### Glycosylation acts positively on the transcription of a specific set of genes

**Fig. 2:**
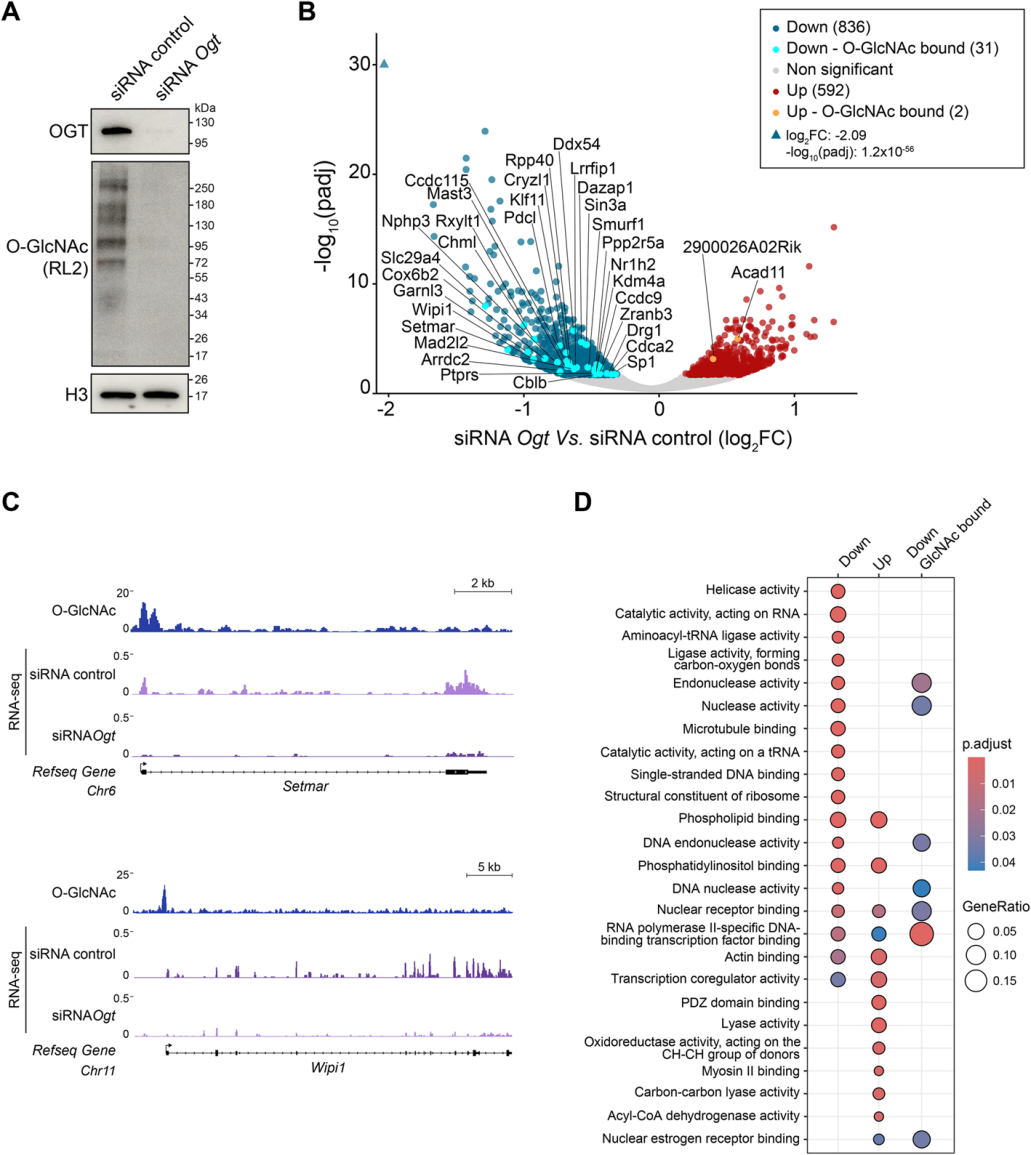
O-GlcNAc is required to sustain the expression of a set of target genes. **A** Western blot detection of OGT, pan-O-GlcNAc (RL2 antibody), and histone H3 (loading control) in total protein extracts from WT ESCs transfected with control siRNA (non-targeting) or with siRNA against *Ogt*. The blots are representative of two independent experiments. **B** Volcano plot showing differential gene expression between ESCs transfected with siRNA control and siRNA anti-*Ogt*. Differentially expressed genes included 836 downregulated (dark blue) and 592 upregulated (dark red) genes (adj. *p*-value < 0.05, Wald Test, any log2FC). Among these, 44 downregulated and 12 upregulated genes have a fold-change higher than two. Thirty-one downregulated (light blue) and two upregulated (orange) genes have an overlapping O-GlcNAc peak in WT cells. **C** Representative genomic tracks of O-GlcNAc regulated promoters as defined by high occupancy of O-GlcNAc-modified proteins in WT cells (top CUT&RUN track) and downregulated upon global removal of O-GlcNAc (bottom RNA-seq track). **D** Gene set enrichment analysis of the up-, down-, and down-O-GlcNAc regulated genes shown in panel B. The number of genes enriched in gene ontology (GO) molecular function (MF) are 1461, 1324, and 31, respectively. The gene ratio reflected by the size of dots indicates the proportion of genes matching a GO set

Having observed a high correlation of RNA Pol II and O-GlcNAc-modified proteins at gene promoters, we then assessed the extent to which the O-GlcNAc modification may regulate their expression. To perturb O-GlcNAc glycosylation, we knocked down the glycosyltransferase *Ogt* by transient transfection of a synthetic small interfering RNA (siRNA). The transfection with *Ogt* siRNA resulted in a strong reduction of OGT protein and global O-GlcNAcylation to nearly undetectable levels (Fig. 2A). Differential gene expression analysis after *Ogt* knockdown identified 836 downregulated and 592 upregulated genes (adj. *p*-value < 0.05, Wald test, any log_2_FC) (Fig. 2B). Differentially expressed genes (DEGs) exhibited a fold-change of less than 2, in agreement with previous functional studies using different *Ogt* perturbation methods [18, 19]. Among these DEGs, 31 downregulated and 2 upregulated genes overlapped with O-GlcNAc peaks in wild-type cells, suggesting a direct role for glycosylation in enhancing their expression (Fig. 2C).

Gene set enrichment analysis (GSEA) revealed that DEGs are enriched for “RNA Pol II specific DNA-binding transcription factors (TFs)” encoding genes, downregulated examples include *Med1*, *Sin3a*, *Med24*, *Atf4*, *Wipi1*, *Asxl1*, and *Sp1* (Fig. 2D). The genes in this class that were either up- or downregulated were distinct from each other (Additional file 3: Table S2). Notably, only the downregulated RNA Pol II-specific TFs had a subset of genes with O-GlcNAc modifications at their promoter regions, indicating that the upregulation of the other TFs of this class may result from indirect secondary effects. Altogether, the data support a model whereby O-GlcNAc enhances the transcription at a small subset of promoter of genes encoding for TFs.

Downregulated O-GlcNAc-occupied promoters also included genes involved in transcriptional co-regulation and helicase activity, supporting the idea that O-GlcNAcylation is necessary to sustain the expression of genes involved in active transcription. Other downregulated direct targets included genes encoding DNA nucleases and endonucleases (*Setmar*,* Aen*,* Dicer1*,* Rad50*,* Mre11a*,* Dclre1c*,* N4bp2*,* Exo1*, and* Zranb3*). The association of the O-GlcNAc modification with DNA repair and other cellular response pathways has been previously documented [49]. In pathological conditions, elevated O-GlcNAc levels were shown to promote cell survival during stress responses induced by environmental cues and human diseases such as cancer and diabetes [50]. Our data therefore suggest that O-GlcNAc may regulate the expression of specific DNA-repair enzymes and endonucleases, potentially serving as a rapid response to stress-induced DNA damage.

### O-GlcNAc is dispensable for RNA Pol II spatial organization in ESCs, but regulates cluster density during differentiation

**Fig. 3:**
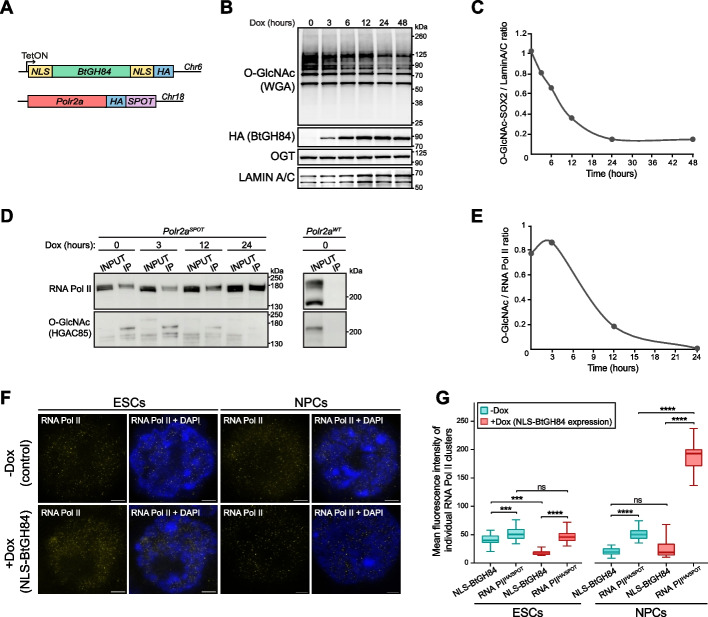
Super-resolution imaging of RNA Pol II clusters after nuclear O-GlcNAc perturbation. **A** Schematic representation of the Tet-ON inducible transgene of bacterial OGA *BtGH84*, fused to a localization peptide (NLS) and the knock-in of the epitope tags HA and SPOT to endogenous *Polr2a* gene encoding RNA Pol II. **B** Western blot (WB) detection of the kinetic depletion of O-GlcNAc (detected by WGA, top), following ectopic expression of the bacterial OGA homolog *BtGH84* fused to a localization peptide, namely *BtGH84-NLS*. The bottom panels show the WB detection of OGT and Lamin A/C (loading control) at the indicated time points following doxycycline induction of *BtGH84-NLS* expression. **C** Quantification by normalized optical density of the WB detection of SOX2-O-GlcNAc at the indicated time points after expression of *BtGH84-NLS*. The blots are shown in Additional file 1: Fig. S2D. **D** Immunoprecipitation of endogenous RNA Pol II using the ESC line described in **A** whereby *Polr2a* was targeted with a knock-in SPOT epitope tag. The immunoprecipitation was performed with magnetic beads coated with anti-SPOT antibodies. The efficiency of RNA Pol II IP and RNA Pol II O-GlcNAc levels were probed by WB analysis at different time points after Dox-induction of *BtGH84-NLS* expression. **E** Quantification of the western blot shown in **D** by the normalized optical density of O-GlcNAc on immunoprecipitated RNA Pol II. The ratio IP RNA Pol II / O-GlcNAc is plotted at different time points after *BtGH84-NLS* expression. **F** Representative micrographs of RNA Pol II and DNA (DAPI) acquired by stimulated emission depletion (STED) microscopy in ESCs and NPCs before and after prolonged nuclear O-GlcNAc depletion by expression of *BtGH84-NLS* (48 h and 5 days, respectively). Scale bars indicate 2 μm. **G** Distribution of fluorescence intensity quantification of RNA Pol II clusters from STED images before and after *BtGH84* induction in ESCs and NPCs (*t*-test. Not-significant (ns): *p* > 0.05; *: *p* < 0.05; **: *p* < 0.01; ***: *p* < 0.001; ****: *p* < 0.0001)

We next investigated whether the O-GlcNAc modification influences RNA Pol II sub-nuclear organization. We used stimulated emission depletion (STED) microscopy to image endogenous RNA Pol II clusters after enzymatic removal of nuclear O-GlcNAc in ESCs and during their differentiation into neuronal progenitor cells (NPCs) (Additional file 1: Fig. S2A) [51]. Pan-O-GlcNAc ChIP-seq experiments showed that cellular differentiation had a negligible impact on the global genomic distribution of O-GlcNAc-modified proteins (Additional file 1: Fig. S2B, C). We refined our O-GlcNAc perturbation by targeting nuclear proteins specifically, through the conditional expression of the bacterial O-GlcNAcase BtGH84 flanked by nuclear localization sequences (NLS) (Fig. 3A) [52]. Expression of the BtGH84-NLS from the ESC genome allowed us to maintain the expression of the transgene during the five days of differentiation protocol. We first established in ESCs the time course of O-GlcNAc removal upon expression of BtGH84-NLS at the global level by western blot detection of O-GlcNAc using the lectin wheat germ agglutinin (WGA) that has a high affinity for O-GlcNAc (Fig. 3B).

To assess the effect of our acute perturbation system on the specific protein, we obtained anti-O-GlcNAc-SOX2 (GlcNAc S248) antibodies—one of the very few available antibodies that recognize the O-GlcNAc-modified state of a specific protein—to measure the kinetics of O-GlcNAc removal on SOX2 specifically (Fig. 3C and Additional file 1: Fig. S2D). We next assessed the efficiency of our acute O-GlcNAc perturbation method on RNA Pol II specifically. To pull down RNA Pol II, we first knocked-in a SPOT epitope tag at the C-terminus of endogenous RNA Pol II in the Tet-ON *BtGH84-NLS* background (Fig. 3A). Immunoprecipitation of RNA Pol II in denaturing condition in untreated cells (without Dox) using anti-SPOT antibodies followed by western blot detection of RNA Pol II and O-GlcNAc (Fig. 3D, 0 h Dox, top and bottom gel, respectively) provided additional evidence of the glycosylated state of RNA Pol II in ESCs. The reciprocal experiment—the pull down of denatured and O-GlcNAc modified proteins followed by western blot detection of RNA Pol II—confirmed the presence of RNA Pol II within the glycosylated nuclear proteome (Additional file 1: Fig. S2E). Next, we estimated the rate of O-GlcNAc removal on RNA Pol II after BtGH84-NLS expression (Fig. 3D, E). The data show that glycosylation of RNA Pol II decreases to undetectable levels after 24 h of BtGH84-NLS expression.

Having validated the BtGH84-NLS enzymatic perturbation method on RNA Pol II, we imaged at super-resolution RNA Pol II clusters, also known as transcription factories [53], after O-GlcNAc removal from nuclear proteins (Fig. 3F). While BtGH84-NLS expression had no detectable impact on RNA Pol II clusters’ fluorescence intensity in ESCs, we observed a significantly higher signal intensity after differentiation to NPCs (*p*-value < 0.0001, unpaired *t*-test; Fig. 3G). Therefore, nuclear hypo-O-GlcNAcylation results in an increased number of RNA Pol II molecules per individual RNA Pol II cluster after differentiation into NPCs. Furthermore, we conclude, from the absence of detectable microscopic phenotype in ESCs, that O-GlcNAc is not required for the control of the size of RNA Pol II clusters in ESCs. The observed higher signal intensity in individual RNA Pol II clusters in NPCs therefore likely results from the combination of hypo-O-GlcNAcylation and chromatin remodeling occurring during cellular differentiation. This result supports the model of O-GlcNAc acting as an antagonist of protein aggregation [17].

### Context-specific RNA Pol II-dependent glycosylation

**Fig. 4:**
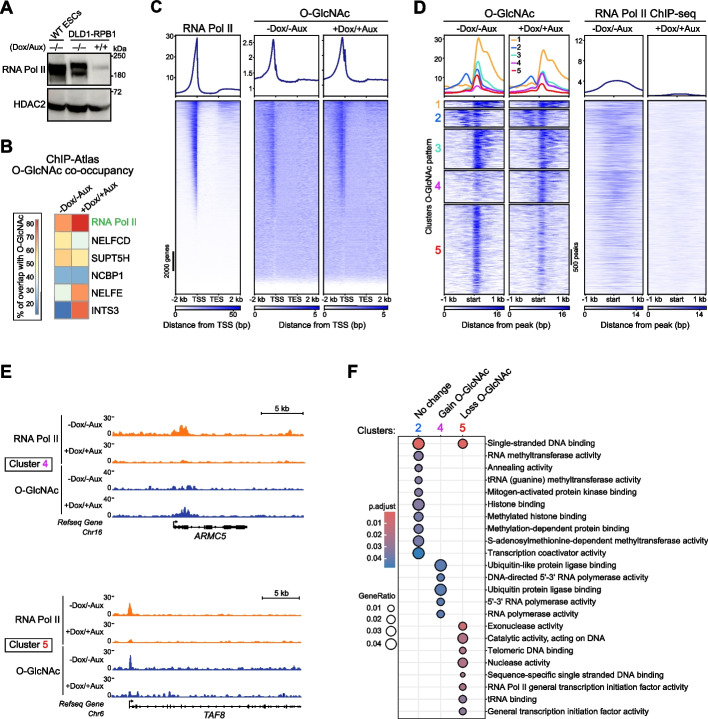
RNA Pol II-dependent O-GlcNAcylation. **A** Western blot showing the TIR1-mediated RNA Pol II degradation in DLD-1 cells upon 14-h Doxycycline/Auxin treatment. **B** Percentages of signal overlap at promoters between 3450 and 2630 O-GlcNAc peaks (before and after Dox treatment, replicate 1) and 2834 ChIP-Atlas datasets in human DLD-1 cells. Proteins previously described to be O-GlcNAcylated are indicated in green. The highest overlap is found with RNA Pol II ChIP-seq GSM5237208. The overlap percentages before and after Dox treatment respectively are RNA Pol II (70/83%), NELFCD (56/39%), SUPT5H (62/55%), NCBP1 (22%), NELFE (40/70%), and INTS3 (12/76%). **C** Genes-stack plots of RNA Pol II ChIP-seq (GEO: GSM5237208, SRA: SRX10580013 [82]) and O-GlcNAc CUT&RUN signals before (middle panel) and after RNA Pol II degradation (right panel). Signal is represented at 21,519 genes (GRCh38 Ensembl) + / − 2 kb. **D** Left: *K*-mean clustering of 6544 O-GlcNAc occupancy peaks (union of replicates), + / − 1 kb around their centers. Five different clusters were defined based on the O-GlcNAc signal before and after RNA Pol II removal by Dox/Aux treatment. Right: Corresponding RNA Pol II ChIP-seq signal (SRA: SRX11070611 and SRX11070613, respectively [54]). **E** Genome browser view illustrating RNA Pol II (SRA: SRX11070611 and SRX11070613 respectively [54]) and O-GlcNAc signal upon Dox/Aux treatment. As an example of cluster 4 (gain of O-GlcNAc), *ARMC5* is shown. To illustrate cluster 5 (loss of O-GlcNAc), the *TAF8* gene is shown. **F** Gene set enrichment comparison using molecular functions for clusters 2 (*n* = 239), 4 (*n* = 347), and 5 (*n* = 751); clusters 1 and 3 did not highlight any significant enrichment. The gene ratio reflected by the size of dots indicates the proportion of genes matching a GO set

The global co-occupancy between RNA Pol II and O-GlcNAc signal density at promoters (Fig. 1E) prompted us to examine the extent by which RNA Pol II glycosylation contributes to the pan-O-GlcNAc CUT&RUN signal. We addressed this question by profiling the genomic occupancy of O-GlcNAcylated proteins before and after rapid RNA Pol II depletion. To this end, we used the previously established RNA Pol II degron system in human DLD-1 colorectal cancer cells [54]. Western blot detection of RNA Pol II confirmed that over 80% of the target protein was degraded after 14 h of treatment with auxin and doxycycline (Aux/Dox, Fig. 4A). Similarly to ESCs, O-GlcNAc proteins primarily occupy active promoters in DLD-1 cells before RNA Pol II degradation (Additional file 1: Fig. S3A, B). Unbiased computational search for co-occupancy with 2,834 DLD-1 ChIP-Atlas datasets identified RNA Pol II as the factor with the greatest genomic overlap with O-GlcNAc (Fig. 4B). Other proteins uncovered with this analysis included the NELF and DSIF complexes, both known to repress RNA Pol II from entering elongation (Fig. 4B) [47, 48]. NCBP1 is a subunit of the Integrator complex that was shown to regulate transcription elongation and initiation [55, 56].

The global correlation between the O-GlcNAc signal and RNA Pol II occupancy across all promoters was even more apparent in DLD-1 cells (Fig. 4C) in comparison with ESCs (Fig. 1E). RNA Pol II degradation resulted in a modest global redistribution of the O-GlcNAc signal around TSSs (Fig. 4C). Further clustering analysis of the O-GlcNAc signal revealed five distinct groups of loci (totaling 6544) whose glycosylation patterns are influenced by RNA Pol II degradation (Fig. 4D, E). These changes in O-GlcNAc patterns can be categorized into three main groups: (1) Clusters 1–2–3 (284, 584, and 1490 peaks, respectively) show no substantial changes in O-GlcNAc occupancy, hence their O-GlcNAc signal is independent of RNA Pol II presence. Genes in cluster 2 encode proteins involved in histone methylation and transcription coactivator activity (Fig. 4F and Additional file 4: Table S3). The set of promoters comprising cluster 5 (2986 peaks) loses O-GlcNAc after RNA Pol II degradation, thus the O-GlcNAcylation of bound proteins depends on RNA Pol II or RNA Pol II itself is the main contributor to the O-GlcNAc signal. GSEA of cluster 5 identifies an enrichment for genes associated with transcription initiation, including *Gtf2e1*, *Taf6*, *Snapc2*, *Taf5*, *Taf12*, *Taf11*, *Taf8*, and *Taf1*. Unexpectedly, this analysis also uncovered a set of promoters whose O-GlcNAc level increases upon RNA Pol II depletion (cluster 4; 1200 peaks). These promoters encode proteins specifically associated with RNA Polymerase II activity such as *Prim1*, *Polr3c*, *Polr1a*, *Polr3g*, and *Polr1d* (Fig. 4F and Additional file 4: Table S3). Their nascent RNA production significantly decreases upon rapid RNA Pol II degradation (Additional file 1: Fig. S3C). The increased O-GlcNAc modification may reflect either the recruitment of O-GlcNAc-modified proteins upon loss of RNA Pol II at these promoters, or a higher glycosylation state of the proteins already bound. Candidates for such factors include NELFE and INTS3, which show an increased overlap with O-GlcNAc in RNA Pol II depleted cells (Fig. 4B, 1.8- and 6.3-fold increase, respectively).

Altogether, the data reveal that depletion of RNA Pol II leads to changes of the O-GlcNAc levels at thousands of promoters, hence uncovering that local glycosylation is dependent on RNA Pol II or the process of transcription.

## Discussion

A prerequisite to the understanding of the function of glycosylation on mammalian chromatin is to acquire knowledge of the genomic occupancy of O-GlcNAc-modified proteins. The lack of available antibodies against specific glycosylated DNA-binding proteins poses a major obstacle to this scientific endeavor. Moreover, the requirement of the O-GlcNAc modification for cell viability in mammals makes any functional investigation particularly difficult. To our knowledge, the genomic profiling of any specific O-GlcNAcylated factor has not yet been determined. Here, we partially circumvented this problem by combining pan-O-GlcNAc genomic profiling and bioinformatic screens for factor co-occupancy.

Our genomic profiling revealed that glycosylated proteins in ESCs are primarily bound to two types of sequences that displayed opposite transcriptional status, namely retrotransposons (silenced) and promoters of cellular genes (expressed). This observation indicates that the local effect of O-GlcNAc modification on transcription is context-dependent and likely depends on the nature of the target proteins. Supporting this idea, O-GlcNAc has been shown to be required for the repressive activity of the repressor Polyhomeotic [16], and in a different context for the function of the activator TF OCT4 [9, 16].

The identity of the O-GlcNAc-modified proteins that enhance or repress transcription in mammalian cells remains a salient point of intrigue. Through a large-scale unbiased *in silico* screen for genomic co-occupancy, we uncovered dozens of DNA-binding proteins that colocalize with O-GlcNAc domains (Figs. 1D and 4B). RNA Pol II and some of its associated factors strongly overlapped with the O-GlcNAc peaks. The CTD’s 52 heptapeptide repeats that contain the O-GlcNAcylated Ser2, Thr4, and Ser5 likely increase the number of O-GlcNAc molecules covalently linked to RNA Pol II. We speculate that the repeated nature of the CTD could amplify the CUT&RUN O-GlcNAc signal contributed by RNA Pol II and therefore favors the observed correlation (Figs. 1E and 4C). The relative position of the RNA Pol II and pan-O-GlcNAc peaks is consistent with the model of O-GlcNAc role in regulating the pre-initiation complex (PIC) formation [24, 26]. This model posits that OGT and PIC-associated kinases act as co-regulators of early transcription and that OGA interacts with elongation factors [27]. The central localization of O-GlcNAc peaks relative to RNA Pol II positioning (Fig. 1F) is consistent with a possible turnover of the O-GlcNAc modification on transcribing RNA Pol II and with the antagonistic relationship between O-GlcNAcylation and phosphorylation at the CTD [57–59]. This observation is supported by the study of Lewis et al. [24] that shows that RNA Pol II is recruited to promoters in its hypophosphorylated form (RNA Pol IIa), glycosylated during PIC formation, and reversed to RNA Pol IIa during transcription initiation concomitantly with Ser5 phosphorylation.

The pleiotropic nature of O-GlcNAc poses a challenge to the interpretation of the molecular phenotypes resulting from acute de-O-GlcNAcylation (Fig. 2B) because it is not possible to untangle direct from indirect effects. The likely direct target genes, as judged by O-GlcNAc occupancy in wild-type, are almost all downregulated and are enriched for transcription factors and DNA modifying enzymes (Fig. 2D). These direct target genes are surprisingly few (*n* = 31), considering the strong correlation genome-wide between RNA Pol II occupancy and O-GlcNAc levels (Figs. 1E, 4 and C). We suggest two hypotheses that could explain this observation: recent evidence revealed that the CTD of RNA Pol II is largely dispensable for transcription [60], hence the lack of O-GlcNAc on the CTD is not expected to cause important transcriptional changes. Alternatively, the O-GlcNAcylation of promoter-bound proteins might be a consequence rather than a cause of transcription. The observed gain of O-GlcNAc at promoters upon degradation of RNA Pol II (Fig. 4D–F), provides indirect support for the latter hypothesis. Although the mechanism remains elusive, our results indicate that the cross-talk between O-GlcNAc and RNA Pol II is a two-way street.

## Conclusions

Our functional genomic approach identifies the genomic context of chromatin occupied by glycosylated proteins in mammalian cells and reveals a context-dependent reciprocal interplay between O-GlcNAc and RNA Pol II.

## Methods

### Cell culture

Murine A9 wild type and genetically engineered ESC lines were cultured in a humidified atmosphere at 37 °C and 6% CO_2_ and grown on gelatin-coated plates in different media: t2i/L media consisted in NDiff (N2B27) (Takara#Y40002), titrated 2i (0.2 μM PD0325901 and 3 μM CHIR 99021, Sigma-Aldrich), 1000 U/mL leukemia inhibitory factor (LIF), 1% FBS (Gibco), 1% penicillin streptavidin (Thermo Fisher Scientific #15,140,122); 2i/L media consisted in 50% NDiff (N2B27) (Takara #Y40002), 50% DMEM/F-12 (Gibco #11,320,033), 1% FBS (Gibco), 1 mM Sodium Pyruvate (Sigma-Aldrich #S8636), 0.1 mM Non-Essential AAs (Sigma-Aldrich #M7145), 2 mM L-Glutamine (Sigma-Aldrich #G7513), 1% penicillin streptavidin (Thermo Fisher Scientific #15,140,122), 0.1 mM β-Mercaptoethanol (Thermo Fisher Scientific #31,350,010); full 2i (1 μM PD0325901 and 3 μM CHIR 99021, Sigma-Aldrich), 1000 U/mL leukemia inhibitory factor (LIF); Serum t2i/L consisted in KO DMEM (Thermo Fisher Scientific#10,829,018) or DMEM (Thermo Fisher Scientific #10,564,011, Thermo Fisher Scientific high glucose #11,995,065 or low glucose #11,885,084), 15% FBS (Gibco), 0.1 mM Non-Essential AAs (Sigma #M7145), 2 mM L-Glutamine (Sigma-Aldrich #G7513), 1% penicillin streptavidin (Thermo Fisher Scientific #15,140,122), 0.1 mM β-Mercaptoethanol (Thermo Fisher Scientific #31,350,010), 1000 U/mL leukemia inhibitory factor (LIF), titrated 2i (0.2 μM PD0325901 and 3 μM CHIR 99021, Sigma-Aldrich); serum/LIF consisted in DMEM (Thermo Fisher Scientific #41,965,039), 15% FBS (Gibco), 0.1 mM Non-Essential AAs (Sigma #M7145), 2 mM L-Glutamine (Sigma-Aldrich #G7513), 1% penicillin streptavidin (Thermo Fisher Scientific #15,140,122), 0.1 mM β-Mercaptoethanol (Thermo Fisher Scientific #31,350,010), 1000 U/mL LIF. The medium was changed daily and cells were passaged every 2–3 days via dissociation in TrypLE Express Enzyme (Thermo Fisher Scientific #12,604,039). Mycoplasma-free status of the cell cultures was verified periodically.

Human colon adenocarcinoma DLD-1 cells expressing OsTIR and with a cassette encoding mini-AID (mAID) and fluorescent protein mClover (mAID + mClover) at the initiation site of the endogenous *RPB1* gene locus (*POLR2A*) [61] were grown in RPMI 1640 medium (Thermo Fisher Scientific #A1049101) supplemented with 10% FBS (Gibco).

### Plasmid construction and cloning

To clone pA/G MNase the pETM14 vector was digested with HindIII restriction enzyme (New England Laboratories #R3104L) according to the manufacturer’s instructions. Protein A/G (pA/G) sequence was ordered as gBlock from Integrated DNA technologies (IDT) and assembled with the digested pETM14 vector using the NEBuilder HiFi DNA Assembly Master Mix (New England Laboratories #E2621L). Purified plasmid from transformed bacteria was sent to PEPCore EMBL Heidelberg Facility for protein expression and purification from Bl21 DE3 competent cells.

### Cell line generation

Clonal ESC lines for Tet-ON expression of *2loxP-3XNLS-BtGH84* and *2loxP-3XNLS-BtGH84*^*D242A*^ were generated as previously described [21]. Briefly, the DNA sequence of *3XNLS-BtGH84* and *3XNLS-BtGH84*^*D242A*^ (Addgene #194,469 and 194,470, respectively) was cloned into the 2loxP vector using the NEBuilder HiFi DNA Assembly reaction (New England Laboratories #E2621). The plasmids were nucleofected using the P3 Primary Cell 4D X LONZA Kit (LONZA #V4XP-3024) in A2loxP-Cre cells. *Cre* expression was induced by exposing the cells to 1 μg/mL of Doxycycline (Sigma-Aldrich #3447) for 24 h. The cells were then cultured in the presence of 350 μg/mL G418 (Sigma Aldrich #G8168-50ML) for 10 days. Positive clones were selected after single clone isolation and genotyping for the knock-in sequence.

To generate the RNA Pol II^HA−SPOT^ knock-in, *3XNLS-BtGH84* ESCs were engineered using the CRISPR/CAS9 technology. The single guide RNA (sgRNA) with the knock-in sequence (*Pol2ra* crRNA 5′−3′: ATGAGGAGAACTGAGCGAAC, *Pol2ra* HA-SPOT tag ssDNA 5′−3′: CAGCCTCACCAGCCCAGCCATCAGCCCAGATGACAGCGATGAGGAGAACGGCGGTGGAGGCAGTTTGGAAGTCCTCTTCCAGGGACCATATCCCTATGACGTCCCAGATTACGCCCCAGACCGCGTAAGAGCTGTTTCTCATTGGAGTTCCtaaGCGAACAGGGCGAAGAGCTGGTTAGGGTCAGACAACCTCGGTGGCC) was designed with homology arms for the interested endogenous gene and nucleofected in mESCs grown in T2i medium on gelatin-coated plates, together with the crRNA, tracrRNA and purified CAS9. Single clones were isolated, expanded in T2i medium and screened by genotyping for positive clones for the knock-in sequence.

### Embryonic stem cell differentiation to neural progenitor cells

Differentiation of ESCs to neuronal progenitor cells (NPCs) was performed as previously described [51]. Briefly, ESCs were seeded in Basal Medium supplemented with 2i/LIF at a density of 20,000 cells/cm^2^ on gelatinized-coated plates and cultured for 12 h to allow for cell attachment. Differentiation was induced by replacing the medium with Basal Medium supplemented with 10 ng/mL of recombinant human bFGF (R&D #233-FB); cells were maintained for 72 h with daily medium exchange. Medium was then replaced with Basal Medium supplemented with 500 nM SAG (Sigma #566,661); cells were maintained for 48 h with daily medium exchange until NPCs collection.

### Protein extraction and western blotting

Cells were detached with TrypLE (Thermo Fisher Scientific #12,604,039), washed with PBS 1X, and pelleted for 5 min at 1000 RPM. For nuclear proteins of Fig. 3D, nuclei were extracted by resuspending the cell pellet in buffer A (10 mM HEPES pH 7.65, 1.5 mM MgCl_2_, 10 mM KCl, 0.5 mM DTT, 1X cOmplete Mini EDTA-free protease inhibitors (Roche)) then incubated for 15 min at 4 °C under gentle rotation. Nuclei were released from cells with the Dounce homogenizer, and pelleted at 250 g for 5 min at 4 °C. Nuclei were washed in buffer N (15 mM HEPES pH 7.65, 10 mM MgCl_2_, 0.5 mM DTT, 250 mM sucrose, 1X cOmplete Mini EDTA-free protease inhibitors (Roche)) and pelleted at 2,800 g for 10 min at 4 °C. For nuclear proteins (Additional file 1: Fig. S2D), nuclei were extracted using the Subcellular Protein Fractionation Kit for Cultured Cells #78,840.

Cells or nuclei were lysed by resuspending the pellets in RIPA Buffer (150 mM NaCl, 1% IGEPAL, 0.5% sodium deoxycholate, 0.1% SDS, 50 mM Tris, pH 8.0) containing 1X cOmplete Mini EDTA-free protease inhibitors (Roche) and incubated 5 min on ice. Genomic DNA was digested using Universal Pierce Nuclease 250 U (Thermo Fisher Scientific #88,702) at 37 °C for 5 min and insoluble chromatin was removed by centrifugation for 1 min at 4 °C at 18,000 g. Protein concentration was determined using the Pierce BCA Protein Assay Kit (Thermo Fisher Scientific #23,227). Equal amounts of proteins were loaded on 4–20% Tris–Glycine gel (NuPAGE), or 4–12% Bis–Tris gel (NuPAGE) for protein detection. Protein dry transfer was performed on 0.2 μm nitrocellulose membranes (BioRad #1,704,159) using a Trans-Blot Turbo Transfer System (Bio-Rad). Membranes were blocked for 1 h in Saturating Buffer (5% BSA, 0.1% Tween-20, PBS 1X) and incubated overnight at 4 °C in primary Antibody Buffer (5% BSA, 0.1% Tween-20, PBS 1X) with primary antibodies (Additional file 5: Table S4). The membranes were washed three times for 5 min at room temperature (RT) in Washing Buffer I (0.5% Triton X-100, 0.5 M NaCl, PBS 1X), one time for 10 min at RT in Washing Buffer II (0.5 M NaCl, PBS 1X), one time for 15 min at RT in PBS 1X and incubated with HRP-conjugated secondary antibodies for 1 h at room temperature. Membranes were washed again as previously described and signal was revealed using the ECL-Prime Western Blot System (Sigma #RPN2232) on an Amersham ImageQuant 800 system.

### *Ogt* siRNA

E14 WT ES cell lines were grown in Serum/LIF media without penicillin streptavidin on a 6-cm dish. Before transfection, 5 μl of Lipofectamine 2000 (Invitrogen #11,668–019) were mixed with 250 μl of Opti MEM reduced Serum Medium (Gibco #51,985–034) and incubated for 5 min at RT. Meanwhile, 10 μl of 10 μM *Ogt* siRNA (Santa Cruz Biotechnology #sc-40781) were mixed with 250 μl of Opti-MEM and then combined with the Lipofectamine2000-Opti-MEM mix to make a transfection mix and incubated for 20 min at RT. Cells were detached with TrypLE and counted to plate 300 × 10^5^ cells/well. Five hundred microliters of transfection mixture were mixed with the cell suspension and the solution was transferred to a pre-gelatinized 6-cm diameter dish. In parallel, a negative control was prepared following the same conditions but using a non-targeting siRNA (Santa Cruz Biotechnology #sc-37007), and a negative control reaction without siRNA was also processed. Cell culture medium was changed 12 h after the experiment and the cells were collected 48 h after siRNA transfection.

### CUT&RUN-seq

Cleavage under targets and release using nuclease (CUT&RUN) was performed as previously described on live nuclei [28], with the modification of the addition of Triton X-100 at different steps of nuclei treatment to increase the nuclei permeabilization as specified below:

The DNA sequence coding for pA/G MNase was synthesized as a gBlock (IDT) and cloned into a pETM14 vector for bacterial expression. The recombinant Protein A/G was purified by EMBL PepCore.

For each condition, 1 × 10^6^ cells were collected and centrifuged at 600 g for 3 min at 4 °C. Nuclei were extracted by resuspending the cell pellet in 1 mL of Nuclear Extraction Buffer (20 mM HEPES–KOH pH 7.9, 10 mM KCl, 0.5 mM Spermidine, 0.1% Triton X-100, 20% Glycerol, 1X cOmplete Mini EDTA-Free Protease Inhibitors (Roche)). Twenty-five microliters of Concanavalin A magnetic beads (Bangs Laboratories #BP531) previously washed twice in Binding Buffer (20 mM HEPES–KOH pH 7.9, 10 mM KCl, 1 mM CaCl_2_) were gently added to each nuclei sample and incubated for 10 min at RT under gentle rotation. The bead-bound nuclei were isolated on a magnetic stand and beads were blocked with 1 mL of Blocking Buffer (20 mM HEPES pH 7.5, 150 mM NaCl, 0.5 mM Spermidine, 0.1% BSA, 2 mM EDTA, 1X cOmplete Mini EDTA-Free Protease Inhibitors (Roche)) for 5 min at RT. The bead-bound nuclei were isolated on the magnetic stand, washed with 1 mL of Wash Buffer (20 mM HEPES pH 7.5, 150 mM NaCl, 0.5 mM Spermidine, 0.1% BSA, 1X cOmplete Mini EDTA-Free Protease Inhibitors (Roche)), incubated for 5 min with 1 mL of Wash Buffer supplemented with 0.1% of Triton X-100, subsequently washed twice with Wash Buffer and resuspended in 300 μL of Antibody Buffer with 0.01% TritonX-100 supplemented with 1 μg of antibody. Samples were incubated under gentle rotation overnight at 4 °C, washed twice with 1 mL of Wash Buffer, and resuspended in 300 μL of Wash Buffer with 0.01% TritonX-100 supplemented with 700 ng/mL of pA/G MNase. The bead-bound nuclei were incubated for 1 h at 4 °C under gentle rotation and washed twice with Wash Buffer. The samples were resuspended in 50 μL of Wash Buffer and placed in iced water to pre-cool to 0 °C. pA/G MNase targeted digestion was initiated by adding 2 μL of CaCl_2_, samples were mixed by flicking and incubated for 30 min on iced water at 0 °C. Digestion reaction was stopped by adding 50 μL of 2X Stop Buffer (200 mM NaCl, 20 mM EDTA, 4 mM EGTA, 1% IGEPAL, 1 mM MnCl_2_). Samples were incubated at 37 °C for 15 min to release the CUT&RUN fragments from the insoluble nuclear chromatin and centrifuged at 16,000 g for 5 min at 4 °C. Supernatants were collected after magnetic beads separation on a magnetic stand and transferred to new tubes. Two microliters of 10% SDS and 2.5 μL of 20 mg/ml of Protein K were added and samples were incubated at 70 °C for 10 min. DNA fragments were purified and size selected using SPRIselect magnetic beads (Beckman Coulter #B23318) following the manufacturer’s protocol for single selection to purify fragments higher than 100 bp. DNA fragments were eluted in 30 μL of 0.1 M TE. Samples concentrations were verified using Qubit III and libraries prepared with NEBNext Ultra II DNA Library Prep Kit for Illumina (E7645S) following the manufacturer’s protocol. Libraries were size selected from Nusieve 3:1 Agarose gel to isolate fragments from 250 base pair (bp) to 460 bp and DNA purified using Monarcharch DNA Gel Extraction Kit Protocol (New England Laboratories #T1020). Libraries quality was evaluated using Tape Station DNA HS D1000 Kit on a Tape Station system (Agilent 4150) and single-end sequenced (SE75) with Illumina NextSeq 500 platform.

### Chromatin immunoprecipitation and sequencing

Cells were detached with TrypLE, washed with PBS 1X, and collected to a final number of 30 × 10^6^ for each replicate. Cells were resuspended in 45 mL PBS 1X and 3.5 mL of Fixing Solution (50 mM HEPES–KOH pH 7.5, 100 mM NaCl, 1 mM EDTA pH 8.0, 0.5 mM EGTA pH 8.0) and fixed with 1.1% formaldehyde (Sigma-Aldrich #252,549) for 10 min at RT. The fixing reaction was quenched with 125 mM Glycine and cells were washed with PBS 1X at 2000 g for 6 min. Cells were lysed in Lysis Buffer 1 (50 mM HEPES–KOH pH 7.5, 140 mM NaCl, 1 mM EDTA pH 8, 10% Glycerol, 0.5% IGEPAL, 0.25% Triton X-100, cOmplete Mini EDTA-Free Protease Inhibitors (Roche)), and washed in Lysis Buffer 2 (10 mM Tris–HCl pH 8, 200 mM NaCl, 1 mM EDTA pH 8, 0.5 mM EGTA pH 8, protease inhibitors). Soluble chromatin was sheared by sonication in Sonication Buffer (50 mM HEPES pH 7.5, 140 mM NaCl, 1 mM EDTA pH 8.0, 1 mM EGTA pH 8.0, 1% Triton X-100, 0.1% C_24_H_40_O_4_, 0.1% SDS, cOmplete Mini EDTA-Free Protease Inhibitors (Roche)) in 1 mL Covaris tubes (Covaris #520,081) using a Covaris 220 system to a chromatin average size of 200–250 bp. Soluble chromatin was separated by collecting the supernatant after centrifugation at 18,000 g for 2 min at 4 °C. Sixty μg or 100 μg of chromatin were immunoprecipitated overnight at 4 °C with 10 μg of antibody. Samples were incubated with 50 μL of Dynabeads protein G magnetic beads (Life Technologies), previously blocked with 0.5% BSA for 4 h at 4 °C. Bead-bound chromatin was washed once with 1 mL of Sonication Buffer, twice with 1 mL of Sonication Buffer supplemented with 500 mM NaCl, twice with 1 mL of Sonication Buffer supplemented with 1 M NaCl and once with 1 mL of LiCl Wash Buffer (20 mM Tris–HCl pH 8.0, 1 mM EDTA pH 8.0, 250 mM LiCl, 0.5% NP-40, 0.5% C_24_H_40_O_4_). Beads-bound chromatin was eluted twice for 15 min at 65 °C in Elution Buffer (50 mM Tris–HCl pH 8.0, 10 mM EDTA, 1% SDS) and de-crosslinked overnight at 65 °C. Chromatin was treated with 0.2 mg/mL of RNaseA at 37 °C for 1 h and with 0.2 mg/mL of Proteinase K at 55 °C for 30 min. DNA was isolated by phenol/chloroform extraction followed by ethanol precipitation and resuspended in 30 μL of 0.1 M TE. Libraries were prepared with NEBNext Ultra II DNA Library Prep Kit for Illumina (New England Laboratories #E7645S) following the manufacturer’s protocol. Libraries were size selected by electrophoresis on Nusieve 3:1 agarose gel to isolate fragments from 250 to 460 bp and DNA purified using Monarcharch DNA Gel Extraction Kit Protocol (New England Laboratories #T1020). The libraries’ quality was evaluated using Tape Station DNA HS D1000 Kit on a Tape Station system (Agilent 4150) and single-end sequenced with Illumina NextSeq 500.

### Total mRNA-seq

For RNA-seq of OGT knock-down WT ESCs, cells were washed 3 times with PBS 1X, centrifuged at 200 g for 5 min, resuspended in 1 mL of TRIzol reagent (Invitrogen) for each cell pellet, and the cell lysate was stored at − 80 °C Samples containing 1 µg total RNA as the starting concentration were used and processed for DNase treatment. rRNA depletion was conducted using the NEBNext rRNA Depletion Kit v2 (NEB #E7400L) and sample libraries were prepared using the NEBNext Ultra II Directional RNA Library Prep Kit for Illumina (NEB #E7760S). The quality of cDNA generated was tested on a Tape Station RNA HS D1000 Kit on a Tape Station system (Agilent 4150) to ensure RNA integrity number (RIN) > 8.5. Messenger RNA libraries and RNA sequencing were performed with pair-ends (PE40) sequencing mode with the Illumina NextSeq 500 platform.

### RNA Pol II Immunoprecipitation

Immunoprecipitation (IP) of endogenous RNA Pol II was performed from RNA Pol II-HA-SPOT cell line grown in KO DMEM Serum/LIF T2i using anti-SPOT antibodies (Chromotek #etd-20). For each IP, 100 × 10^6^ cells were detached with TrypLE, washed twice with PBS 1X, and collected at 1500 g for 5 min. Cell pellet was resuspended in 7 mL of Buffer A (10 mM HEPES pH 7.65, 1 mM MgCl_2_, 10 mM KCl, 0.5 mM DTT, 1X cOmplete Mini EDTA-Free Protease Inhibitors) and incubated on ice for 15 min. Nuclei were extracted using the Dounce homogenizer, volume was equilibrated to 10 mL with buffer A, and nuclei were collected by centrifugation at 300 g for 10 min at 4 °C. The supernatant, which corresponds to the cytoplasmic fraction, was discarded and nuclear pellet was washed with 10 mL of Buffer N (40 mM HEPES pH 7.65, 1 mM MgCl_2_, 0.5 mM DTT, 250 mM Sucrose, 1X cOmplete Mini EDTA-Free Protease Inhibitors) and centrifuged at 1000 g for 10 min at 4 °C. The nuclear pellet was resuspended in 400 μL of Buffer N and nuclei were lysed by adding 400 μL of 2X Lysis Buffer (0.5% SDS). Genomic DNA was digested by adding 8 μL of Universal Pierce Nuclease and by incubating the samples for 10 min at 37 °C. Proteins were denatured at 95 °C for 2 min and insoluble debris removed by collecting the supernatant after centrifugation at 13,000 g for 5 min at 4 °C. Protein concentration was estimated using the BCA Protein Assay Kit as previously described and an equal amount of nuclear protein extract was used for each IP. 100 μL of Spot-Trap Magnetic particles M-270 (Chromotek #etd-20) were washed twice with 1 mL of beads Washing Buffer (10 mM HEPES pH 7.65, 1 mM CaCl_2_, 0.01% NP-40, 0.1% bovine serum albumin (BSA)) and resuspended in 50 μL of Washing Buffer (20 mM HEPES pH 7.65, 250 mM NaCl, 1 mM CaCl_2_, 1 mM MgCl_2_, 0.5% IGEPAL, 1X cOmplete Mini EDTA-Free Protease Inhibitors). Three volumes of 3/4X Binding Buffer (66 mM HEPES pH 7.65, 333 mM NaCl, 1.4 mM CaCl_2_, 2 mM MgCl_2_, 1X cOmplete EDTA-Free Mini Protease Inhibitors) and 50 μL of magnetic beads were added to the nuclear extract and sample incubated overnight at 4 °C. Bead-bounded proteins were washed 4 times for 10 min with 1 mL of Washing Buffer and eluted twice for 5 min at 95 °C in 50 μL of Elution Buffer (20 mM HEPES pH 7.65, 500 mM NaCl, 1% SDS, 1% IGEPAL, 0.5% Sodium deoxycholate, 0.5 mM DTT). Immunoprecipitated protein samples were analyzed by total protein staining using SYPRO Ruby Protein Gel Staining (Thermo Fisher Scientific #S12000) and by Western Blot analysis.

### WGA-IP immunoprecipitation

Nuclear protein samples were extracted and prepared as previously described [21]. Two hundred μl of Promega High Magnetic® Streptavidin magnetic beads (Promega #V7820) were washed twice with 2 mL of Beads Washing Buffer (10 mM HEPES pH 7.65, 1 mM CaCl_2_, 0.01% NP-40, 0.1% bovine serum albumin (BSA)), resuspended in 2 mL of Beads Washing Buffer supplemented with 500 μg of Biotin-conjugated Wheat germ agglutinin (WGA) and incubated for 2 h at RT on a rocker. WGA-bound beads were washed with 1 mL of Beads Washing Buffer and resuspended in 50 μl of WGA Washing Buffer (20 mM HEPES pH 7.65, 250 mM NaCl, 1 mM CaCl_2_, 1 mM MgCl_2_, 0.5% NP-40, 1X cOmplete EDTA-Free Mini Protease Inhibitors). Two negative controls were also used: to saturate WGA binding sites and ensure the specificity of O-GlcNAc protein binding, 200 μl of Promega High Magnetic® Streptavidin magnetic beads were incubated in parallel with 1.1 M of free O-GlcNAc powder (Sigma Aldrich #A8625). Purified bacterial BtGH84 protein was also used in parallel as a negative control to treat the nuclear protein extract for O-GlcNAc global removal: 1/2 of the IP sample was eluted at 95° C in 20 mM HEPES pH 7.65, and 2 μl of 50 ng/μl purified bacterial BtGH84 were added to the WGA-IP sample to have a final enzyme quantity of 100 ng. WGA-IP was treated with bacterial BtGH84 for 4 h at 20 °C. WGA-IP sample and negative control samples were analyzed by Western Blot.

### Imaging of RNA Pol II by stimulated emission depletion microscopy

3XNLS-BtGH84 and RNA Pol II^HA−SPOT^ (3XNLS-BtGH84 background) ESCs were plated on glass coverslips (170 μm thickness) at a density of 3 × 10^4^/0.8 cm^2^. For ESCs, cells were plated on the glass coverslips 12 h before the experiment, while for NPCs the cells were plated on glass coverslips when starting the differentiation protocol. Cells were fixed for 20 min at RT with 4% of formaldehyde solution diluted in PBS 1X and permeabilized for 5 min at RT with 0.3% Triton X-100 solution diluted in PBS 1X. Cells were washed three times for 5 min with 1X PBS and blocked for 30 min at RT with PBT solution (1X PBS, 0.1% Tween-20, 5% BSA). Blocking solution was removed and cells were incubated for 1 h at RT with primary antibody diluted 1:1000 in PBT solution. Primary antibody solution was removed, and cells washed three times with PBS 1X for 5 min at RT and incubated 1 h at RT with a secondary anti-mouse antibody labeled with STAR RED diluted 1:200 in PBT solution. Secondary antibody solution was removed, and cells were washed twice in PBS 1X for 5 min at RT and incubated for 5 min at RT in PBS 1X supplemented with 1:1000 DAPI solution (Thermo Fisher Scientific #D1306). Cells were washed once with PBS 1X, excess of solution was drained and coverslips were properly mounted with Abberior Mount Solid Antifade (Abberior #MM- 2013-2X15ML). Imaging by stimulated emission depletion (STED) microscopy was performed using a STEDYCON mounted on an upright Zeiss microscope in confocal or STED modes. Samples were imaged with a Zeiss 100 Å ~ 1.46 NA objective, 20 nm pixel size, 5 μs pixel dwell time, 15-line accumulations, and a pinhole of 64 μm. STED laser powers were 100% of the 640 nm and 775 nm 51.73% for the STAR RED channel, whereas for the DAPI channel was 1.8% of the 405 nm laser.

### Bioinformatics analysis

#### CUT&RUN and ChIP-seq data preprocessing

Quality control was done with FastQC [62] v0.11.9: fastqc –outdir $outputfolder –threads $nbcpu –quiet –extract –kmers 7 -f 'fastq' $input.fastq.gz. Adapters and low-quality reads were removed with trim-galore [63] v0.4.3: trim_galore –phred33 –quality 20 –stringency 1 -e 0.1 –length 20 –output_dir./ $input.fastq.gz.

Reads were aligned to mm10 or hg38 with Bowtie [64–66] v2.3.4.1 and the bam were sorted using samtools [67, 68] v1.9. For single-end data: bowtie2 -p $nbcpu -x species/genome/genome -U $input.fastq.gz –sensitive –no-unal 2 > $log | samtools sort -@$nbcpu -O bam -o $output.bam. For paired-end data: bowtie2 -p $nbcpu -x species/genome/genome −1 $input1.fastq.gz −2 $input2.fastq.gz -I 0 -X 500 –fr –dovetail –sensitive –no-unal 2 > $log | samtools sort -@$nbcpu -O bam -o $output.bam. More details for the multi-read alignment are available in the GitHub repository provided below.

Only primary alignments were kept using samtools v1.9: samtools view -o $output.bam -h -b -q 20 -F 0 × 800 $input.bam. Reads not aligned to consensus chromosomes were excluded with samtools v1.9: samtools view -o $output.bam -h -b $input.bam 'chr1' 'chr2' 'chr3' 'chr4' 'chr5' 'chr6' 'chr7' 'chr8' 'chr9' 'chr10' 'chr11' 'chr12' 'chr13' 'chr14' 'chr15' 'chr16' 'chr17' 'chr18' 'chr19' 'chrX' 'chrY'. Chromosomes 20, 21, 22 were added for human. Duplicates were removed with picard [69] v2.18.2: picard MarkDuplicates INPUT = $input.bam OUTPUT = $output.bam METRICS_FILE = $metrics.txt REMOVE_DUPLICATES = 'true' ASSUME_SORTED = 'true' DUPLICATE_SCORING_STRATEGY = 'SUM_OF_BASE_QUALITIES' OPTICAL_DUPLICATE_PIXEL_DISTANCE = '100' VALIDATION_STRINGENCY = 'LENIENT' QUIET = true VERBOSITY = ERROR.

Bigwig files normalized by the genome size (2,308,125,349 for mouse and 2,701,495,761 for human) were generated with deeptools [70] v3.0.2. For single-end data: bamCoverage –numberOfProcessors $NBCPU –bam $input.bam –outFileName $output.bw –outFileFormat 'bigwig' –binSize 50 –normalizeUsing RPGC –effectiveGenomeSize genomesize –scaleFactor 1.0 –extendReads 150 –minMappingQuality '1'. For paired-end data: bamCoverage –numberOfProcessors $NBCPU –bam $input.bam –outFileName $output.bw –outFileFormat 'bigwig' –binSize 50 –normalizeUsing RPGC –effectiveGenomeSize 2,308,125,349 –scaleFactor 1.0 –extendReads –minMappingQuality '1'.

#### RNA-seq data preprocessing and differential expression

FastQC 0.11.9 was used for quality control: fastqc –outdir $outfolder –threads $nbcpu –quiet –extract –kmers 7 -f 'fastq' input.fastq.gz.

Adapters and low quality reads were removed with trim-galore 0.4.3: trim_galore –phred33 –quality 20 –stringency 1 -e 0.1 –length 20 –output_dir./ $input.fastq.gz.

Alignment was performed with STAR [71] 2.6.0b: STAR –runThreadN $nbcpu –genomeLoad NoSharedMemory –genomeDir 'mm10/rnastar_index2/mm10/files' –readFilesIn $input.fastq.gz –readFilesCommand zcat –outSAMtype BAM SortedByCoordinate –outSAMattributes Standard –outSAMstrandField None –outFilterIntronMotifs RemoveNoncanonical –outFilterIntronStrands RemoveInconsistentStrands –outSAMunmapped None –outSAMprimaryFlag OneBestScore –outSAMmapqUnique "255" –outFilterType Normal –outFilterMultimapScoreRange "1" –outFilterMultimapNmax "10" –outFilterMismatchNmax "10" –outFilterMismatchNoverLmax "0.3" –outFilterMismatchNoverReadLmax "1.0" –outFilterScoreMin "0" –outFilterScoreMinOverLread "0.66" –outFilterMatchNmin "0" –outFilterMatchNminOverLread "0.66" –outSAMmultNmax "−1" –outSAMtlen "1" –outBAMsortingBinsN "50".

Counts were obtained with subread [72] v2.0.1: featureCounts -a Mus_musculus.GRCm38.102.chr.gtf -F GTF -o ESCRNAseq_SRR11294181counts.txt -T $nbcpu -s 0 -Q 0 -t 'exon' -g 'gene_id' –minOverlap 1 –fracOverlap 0 –fracOverlapFeature 0 -C input.bam.

Differential expression was performed with DESeq2 [73] v1.22.1.

#### Peak detection

For ChIP-seq and CUT&RUN data, the peak detection was performed in several ways. See the Github code repository for details and parameters. Briefly, Macs2 [74] v2.2.7.1 was used to detect broad peaks (macs2 callpeak -t $input.bam -c $control.bam -n $expname –outdir $outfold -f BAM -g 1.87e9 -s $tagsize –nomodel –extsize 150 –keep-dup $dupthresh –broad –broad-cutoff $qvalue) or narrow peaks (macs2 callpeak -t $input.bam -c $control.bam -n $expname –outdir $outfold -f BAM -g 1.87e9 -s $tagsize -q $qvalue –nomodel –extsize 150 –keep-dup $dupthres). Large H3K27me3 peaks were detected with hiddenDomains [75] v3.1 (hiddenDomains -g $chromInfoFile -b 300 -t $input.bam -c $control.bam -o $outfold). The ATAC-seq peaks were detected from the Galaxy workflow (see repository) using Macs2 v2.1.1.20160309 (macs2 callpeak -t $input.bam –name $expname –format BAMPE –gsize 1.87e9 –keep-dup '1' –qvalue '0.1' –nomodel –extsize '75' –shift '0').

#### Irreproducible discovery rate

The Irreproducible Discovery Rate (IDR) plots (Additional file 1: Fig. S1A, B, S2B, S3A) were generated with IDR v2.0.3 [76] using the command: idr –samples $rep1 $rep2 –input-file-type narrowPeak –rank q.value –output-file $OUTFOLD"prefix" –plot –log-output-file $OUTFOLD"log.txt". The IDR measures the reproducibility of findings by creating a curve, which quantitatively assesses when the findings are no longer consistent across replicates. It compares a pair of ChIP-seq peak ranked lists and then fits the bivariate rank distributions over the replicates to separate signal from noise based on a defined confidence of rank consistency and reproducibility of identifications, i.e. the IDR threshold. The panels given as output are defined as follows: replicate 1 peak ranks versus replicate 2 peak ranks (upper left) and log_10_ transformed (upper right) with peaks that do not pass the specified IDR threshold colored in red. Bottom row panels describe peaks rank versus -log_10_ IDR scores, with overlay box plots displaying the distribution of IDR values in each 5% quantile.

#### Genomic compartments analysis

The analysis for Fig. 1A and Additional file 1 Fig. S1C and S3B were performed using an in-house R package, which code is provided in the below-mentioned GitHub repository. Briefly, each genomic compartment is defined as: Active Promoters: (H3K27ac peaks overlapping TSS − / + 1 kb); Transcription Initiation Sites (TSS − 1/ + 1 kb overlapping with Ser5P peaks); Heterochromatin Domains (H3K9me3 peak intervals); Bivalent promoters (H3K4me3 and H3K27me3 peaks overlapping TSS − / + 1 kb); Polycomb Domains (Suz12 and RING1B peaks overlapping each other); Transcription Elongation Sites (TSS + 1 kb to TES overlapping Ser2P peaks); and Transcription Termination Sites (TES + 50 bp intervals).

#### ChIP-Atlas

Figures 1D and 4B were generated with the Chip-Atlas [32] “Enrichment Tool” querying O-GlcNAc peaks against the mouse mm10 and hg38 databases. Note that the web interface has been updated since this figure was made. All files and codes to reproduce the figure are provided in the GitHub repository.

#### Geneset enrichment analysis

Figures 2D and 4F were generated with the R package ClusterProfiler [77, 78] v4.8.1 and show a “compareCluster” dotplot of the top 10 enrichment terms of the “enrichGO” function using the ontology “MF” (Molecular Function).

#### STED images analysis

High-resolution images were analyzed with Fiji (v2.3.0). Using the images acquired with the DAPI channel, nuclei were isolated from the cell area by modulating the median and threshold values and by generating a binary file. Nuclear SPOT antibody signal acquired with the STAR RED channel was analyzed using the RS-FISH plugin [79] for spot detection. Number of spots, spots mean and max intensity, and spots density parameters on the nuclear area were analyzed on Prism (v 9.3.1) using unpaired *t*-test for statistical analysis.

#### Statistical analyses

The specific statistical tests used are indicated in the text and the corresponding figure legends. Not-significant (ns): *p* > 0.05; *: *p* < 0.05; **: *p* < 0.01; ***: *p* < 0.001; ****: *p* < 0.0001.

## Supplementary Information

Additional file 1: Fig. S1-3. Fig. S1. O-GlcNAc profiling in ESCs. Fig. S2. Nuclear O-GlcNAc perturbation during ESCs' differentiation to NPCs. Fig. S3. O-GlcNAc profiling after RNA Pol II degradation in DLD-1 cells.

Additional file 2: Table S1. References O-GlcNAc modification of proteins with high binding overlap with O-GlcNAc.

Additional file 3: Table S2. Detailed ontologies of Fig. 2D.

Additional file 4: Table S3. Detailed ontologies of Fig. 4F.

Additional file 5: Table S4. List of antibodies used in this study.

Additional file 6: Table S5. List of omics datasets.

## Data Availability

The raw and processed files generated for this study are available at ArrayExpress under the accession numbers: E-MTAB-14307 [83], E-MTAB-14308 [84], E-MTAB-14309 [85], E-MTAB-14310 [86], E-MTAB-14313 [87]. All the files necessary for reproducing the figures are available on Zenodo: https://zenodo.org/records/12793186 [88] and https://zenodo.org/records/13444099 [89]. The summary of all the datasets used in this study is provided in the Additional file 6: Table S5. The code to reproduce the results in this manuscript is available at the GitHub repository: https://github.com/descostesn/chromglycomethods [90].
