# Supplementary material for: Functional genomic profiling of O-GlcNAc reveals its context-specific interplay with RNA polymerase II
Source: Genome Biol. 2025 Mar 24;26:69. doi: 10.1186/s13059-025-03537-2 (PMC11931877; doi:10.1186/s13059-025-03537-2)
Supplement: Supplementary file 1 — Additional file 1: Fig. S1-3. Fig. S1. O-GlcNAc profiling in ESCs. Fig. S2. Nuclear O-GlcNAc perturbation during ESCs' differentiation to NPCs. Fig. S3. O-GlcNAc profiling after RNA Pol II degradation in DLD-1 cells. [file 13059_2025_3537_MOESM1_ESM.pdf]

# Functional genomic profiling of O-GlcNAc reveals its context-specific interplay with RNA polymerase II

Sofia Rucli[1,2,3], Nicolas Descostes[1,3], Yulia Ermakova[1], Urvashi Chitnavis[1], Jeanne Couturier[1], Ana Boskovic[1], Matthieu Boulard[1,*]

[1] Epigenetics & Neurobiology Unit, EMBL Rome, European Molecular Biology Laboratory, Italy

[2] Collaboration for a joint PhD degree between EMBL and Heidelberg University, Germany

[3] These authors contributed equally: Sofia Rucli, Nicolas Descostes

Correspondence: matthieu.boulard@embl.it

**Additional file 1:**

Figures S1 to S3.

**Figure S1.** O-GlcNAc profiling in ESCs (pages 2-3)

**Figure S2.** Nuclear O-GlcNAc perturbation during ESCs' differentiation to NPCs (pages 4-5)

**Figure S3.** O-GlcNAc profiling after RNA Pol II degradation in DLD-1 cells (pages 6-7)

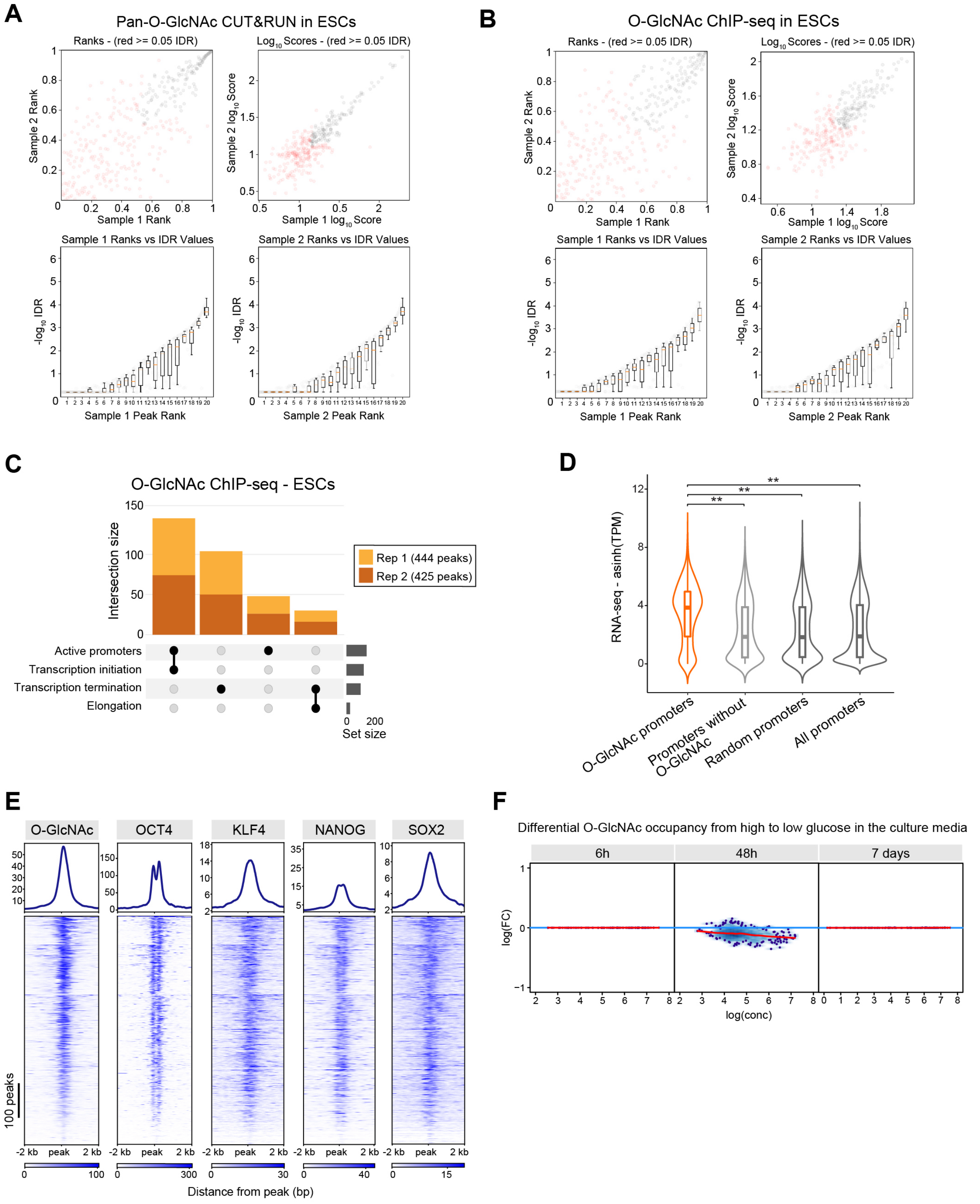


**Fig. S1 O-GlcNAc profiling in ESCs** *(continued on page 3)*

**A** Irreproducible Discovery Rate (IDR) of the two CUT&RUN replicates in mouse ESCs (527/523 peaks) with significant IDR < 0.05 in black. **B** Irreproducible Discovery Rate (IDR) of the two ChIP-Seq replicates in mouse ES cells (444/425 peaks) with significant IDR < 0.05 in black. **C** Upset plot of the location of O-GlcNAc ChIP-seq peaks in mouse ESC (444 and 425 peaks for replicates 1 and 2) in functional genomic compartments: Active promoters (95/101 peaks), Transcription Initiation (83/85 peaks), Transcription Termination (70/68 peaks) and

Transcription Elongation (16/19 peaks). O-GlcNAc preferentially occupies active promoters at the transcription initiation sites. **D** Violin plot showing the expression levels of genes using a publicly available RNA-seq (SRA: SRR11294181) with promoters highly occupied by O-GlcNAc (ChIP-seq) modified proteins (n=236), promoters without detectable O-GlcNAc signal (n=236), randomly selected promoters (n=236), and all promoters (n=21,085). The boundaries of the overlaid box plot show the data above the 1$^{st}$ and within the 3$^{rd}$ quartiles, whiskers indicate minimum and maximum values, the horizontal bar in the box plot shows the median. Statistical comparison was performed using a Mann-Whitney-Wilcoxon two-sided test. *: $p < 0.05$; **: $p < 0.01$. **E** Heatmap of CUT&RUN O-GlcNAc signal and ChIP-seq signal from ChIP-Atlas selected pluripotency factors previously shown to be O-GlcNAcylated, OCT4, KLF4 and SOX2, and NANOG that is not O-GlcNAc modified, +/- 1 kb around the center of O-GlcNAc peaks. The list of peaks was obtained by computing the union of the peak replicate (702 peaks). **F** Differential occupancy analysis of CUT&RUN O-GlcNAc peaks between cells grown in high glucose conditions and cells grown in low glucose for 6 h, 48 h and 7 days.

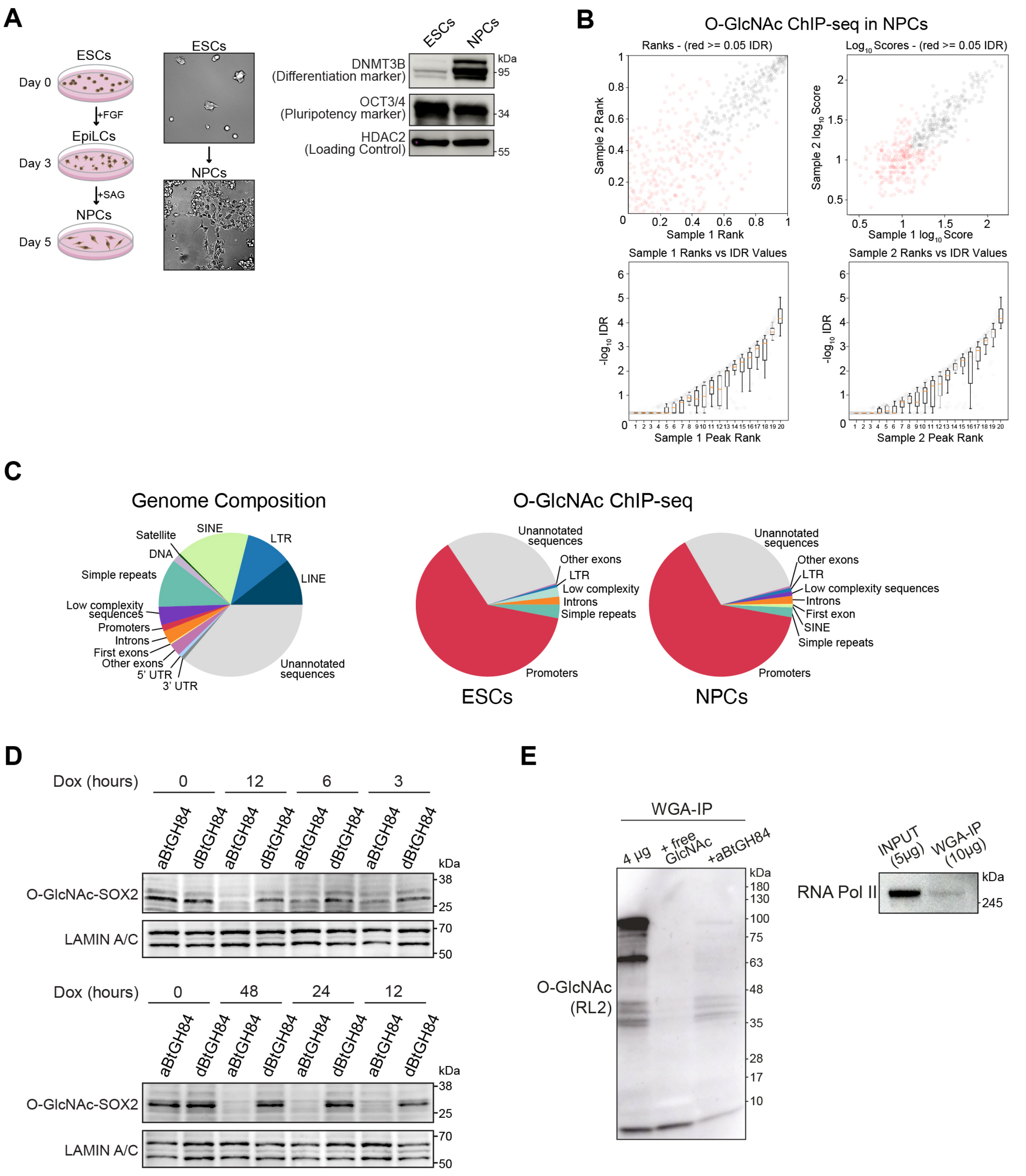


**Fig. S2 Nuclear O-GlcNAc perturbation during ESCs' differentiation to NPCs** *(continued on page 5)*

**A** Left: schematic representation of ESCs to NPCs differentiation protocol. Middle: representative brightfield images of ESCs before differentiation (top) and after differentiation in NPCs (bottom) cells at ESC and NPC stages. Right: Western Blot analysis of ESCs to NPCs differentiation using DNMT3B and OCT3/4 markers. **B** Irreproducible Discovery Rate (IDR) of the two ChIP-Seq replicates in mouse NPCs (640/794 peaks) with significant IDR < 0.05 in black. **C** Pie-chart showing the O-GlcNAc ChIP-seq signal distribution at different genomic loci (right) in comparison with the representative proportions of each locus in the mouse genome

(left). O-GlcNAc ChIP-seq was performed in duplicates using O-GlcNAc HGAC85 antibody in ESCs (444/425 peaks) and NPCs (640/794 peaks) and the intersection of each replicate peak was used (329 and 498 peaks). **D** Western blot detection of O-GlcNAc-modified SOX2 for the indicated time intervals following *BtGH84-NLS* induction. **E** Left: WGA-IP of nuclear O-GlcNAcylated proteins confirmed by O-GlcNAc WB analysis, in presence of large excess of free-GlcNAc and in presence of purified BtGH84 enzyme as negative controls. Right: RNA Pol II western blot detection before and after WGA-IP.

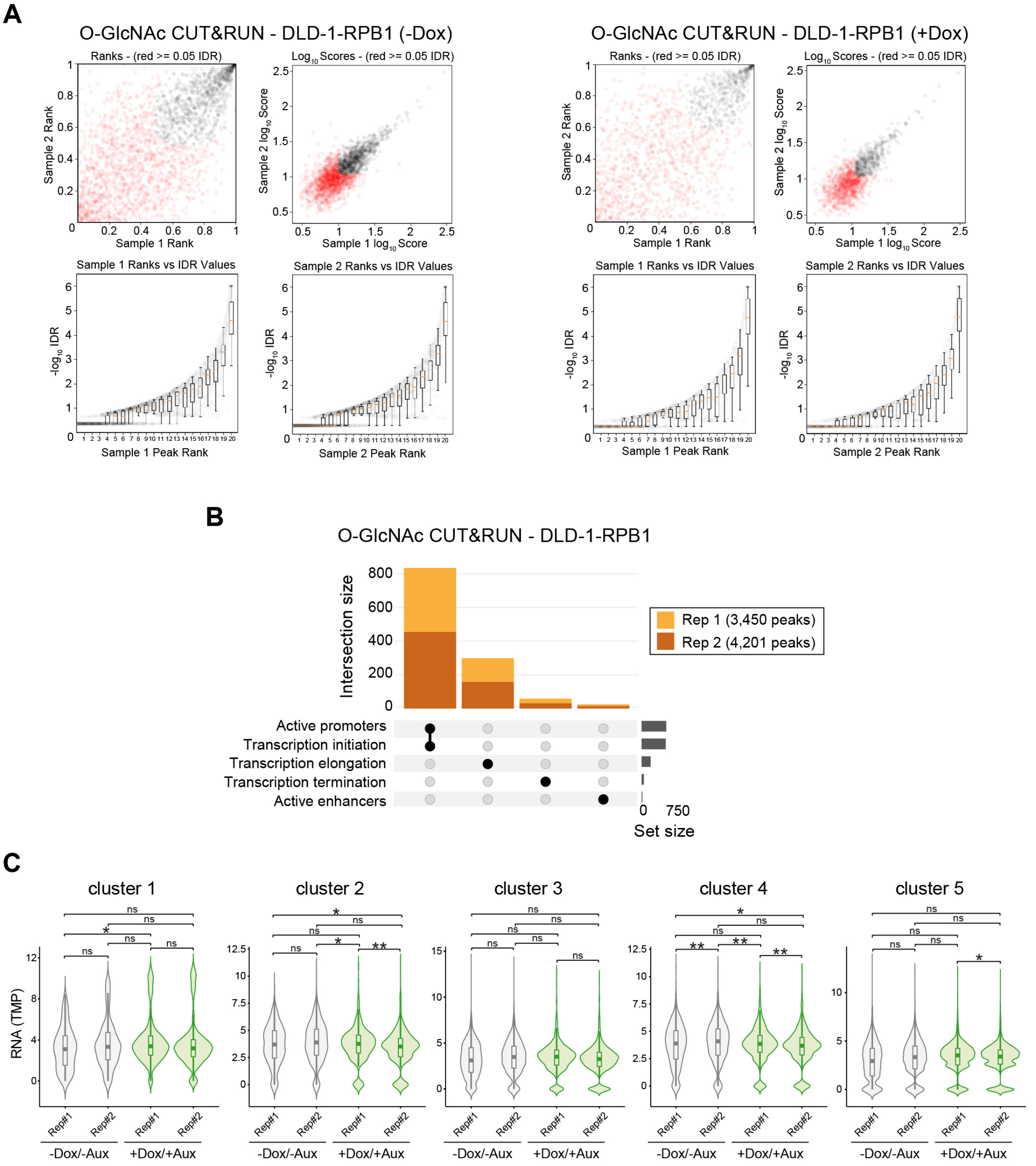


**Fig. S3. O-GlcNAc profiling after RNA Pol II degradation in DLD-1 cells** *(continued on page 7)*

**A** Irreproducible Discovery Rate (IDR) of the two CUT&RUN replicates in human DLD-1 cells before (3,450/4,201 peaks, left panel) and after (2,630/1,848 peaks, right panel) Dox treatment with significant IDR < 0.05 in black. **B** Upset plot of the location of O-GlcNAc CUT&RUN peaks in human DLD-1 cells without Doxycycline/Auxin (3,450 and 4,201 peaks for replicates 1 and 2) in functional genomic compartments: Active promoters (389/466 peaks), Transcription Initiation (381/456 peaks), Transcription Elongation (147/167 peaks), Transcription Termination (32/41 peaks) and active enhancers (10/14 peaks). O-GlcNAc preferentially occupies active promoters at the transcription initiation sites. **C** Violin plot showing gene expression levels after RNA Pol II degradation of the genes forming the 5 clusters shown in

Fig. 4D. The analysis is performed with generated RNA-seq expression data from DLD-1 cells (Mann-Whitney-Wilcoxon two-sided test. Non-significant (ns): $p > 0.05$; *: $p < 0.05$; **: $p < 0.01$).
